# Automated time-resolved tracking algorithm of the atrioventricular plane displacement in CMR images

**DOI:** 10.1186/1532-429X-18-S1-P43

**Published:** 2016-01-27

**Authors:** Felicia Seemann, Ulrika Pahlm, Katarina Steding-Ehrenborg, Ellen Ostenfeld, Håkan Arheden, Marcus Carlsson, Einar Heiberg

**Affiliations:** 1 grid.4514.40000000109302361Department of Clinical Physiology, Skåne University Hospital, Lund University, Lund, Sweden; 2 grid.4514.40000000109302361Department of Health Sciences, Lund University, Lund, Sweden; 3 grid.4514.40000000109302361Department of Biomedical Engineering, Faculty of Engineering, Lund University, Lund, Sweden

## Background

The atrioventricular plane displacement (AVPD) is an indicator for systolic and diastolic function in the left and right side of the heart. AVPD accounts for 70% of the total stroke volume [1]. A reduced AVPD is associated with cardiac disease and aging. Manual measurements is considered reference standard in CMR. However, manual measurements are observer dependent and usually done only in the end diastole and end systole time frames, hence only the total distance can be computed. Time-resolved tracking allow the computation of AVPD as well as velocity of the AV-plane throughout the whole cardiac cycle, yet is time consuming. Automated methods for time-resolved tracking of the AVPD would reduce time consumption. The purpose of this study was to develop and validate a method that automatically track the AV-plane in CMR images, using manually placed points in one time frame as input.

## Methods

The study included 137 subjects who underwent CMR imaging; 32 athletes (16 females), 26 normal subjects (11 females), and 79 patients who suffered from myocardial infarction (13 females). The algorithm is initialized by manually indicating the AV-plane in the end diastolic time frame, using a total of 8 points in long axis CMR images; 3 points in the four chamber view, 3 points in the three chamber, and 2 points in the two chamber. A region around each point is extracted in the current time frame, and the normalized cross correlation between these pixels and the corresponding region in the next time frame is calculated. The maximum of the correlation matrix determines the localization of the point in the next time frame. The method is repeated for each time frame, then the tracked points are filtered by a Kalman filter, and the total movement of AVPD in the left and right heart is computed. Algorithm parameters were optimized using a subset of 16 subjects. Manually placed points in end systole were used as validation. Inter-observer variability of manual measurements was performed on 20 subjects.

## Results

**Figure 1:**
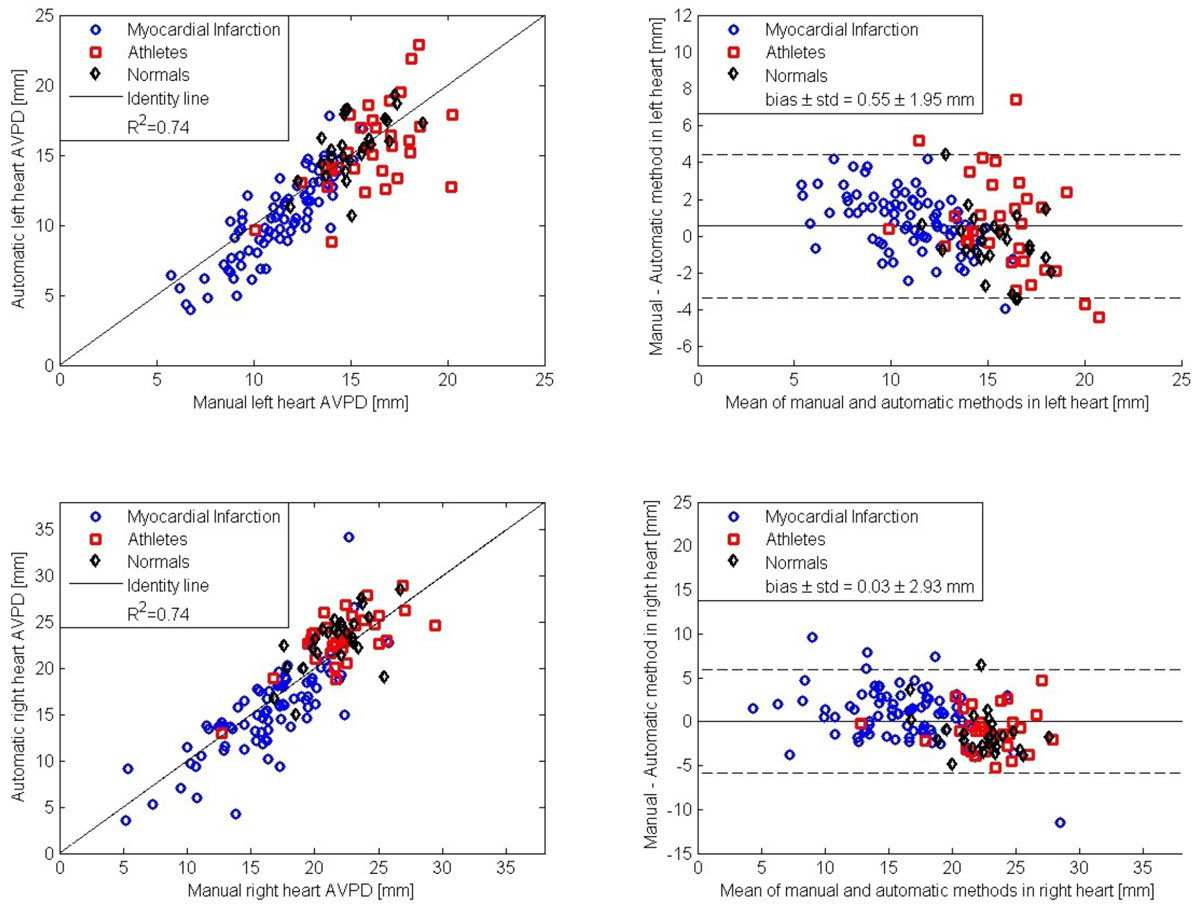
**Left panel shows scatter plots between automatic algorithm and manual measurements (full line is the identity line)**. Right panel shows the bias (full line) according to Bland-Altman (± 2SD, dashed lines) in atrioventricular plane displacement between the algorithm and reference. Top row shows the results for the left heart and bottom row shows the results for the right heart. Blue circles, Myocardial Infarction; Red squares, Athletes; Black diamonds, Normals.

The difference between the proposed algorithm and the reference standard in the left heart was (mean ± SD) 0.55 ± 1.95 mm (R^2^=0.74), and 0.03 ± 2.91 mm (R^2^=0.74) in the right heart, Figure 1. The difference between the proposed algorithm and the reference standard was statistically significant, p < 0.01, on both sides. The inter-observer variability was 0.57 ± 0.68 mm for the left heart and 0.04 ± 2.39 mm for the right heart.

## Conclusions

The proposed algorithm show good agreement and low bias with the reference standard, and with an agreement in parity with inter-observer variability. The algorithm show potential for fully automatic tracking of atrioventricular plane displacement in CMR images.
